# Impact of involvement of non-formal health providers on TB case notification among migrant slum-dwelling populations in Odisha, India

**DOI:** 10.1371/journal.pone.0196067

**Published:** 2018-05-23

**Authors:** Ambarish Dutta, Sarthak Pattanaik, Rajendra Choudhury, Pritish Nanda, Suvanand Sahu, Rajendra Panigrahi, Bijaya K. Padhi, Krushna Chandra Sahoo, P. R. Mishra, Pinaki Panigrahi, Daisy Lekharu, Robert H. Stevens

**Affiliations:** 1 Asian Institute of Public Health, Bhubaneswar, India; 2 Stop TB Partnership, Geneva, Switzerland; 3 WHO-RNTCP Technical Assistance Network, Odisha, India; 4 University of Nebraska Medical Centre, Omaha, Nebraska, United States of America; 5 Independent consultant, Geneva, Switzerland; Stanford University School of Medicine, UNITED STATES

## Abstract

**Background:**

Migrant labourers living in the slums of urban and industrial patches across India make up a key sub-population so far controlling Tuberculosis (TB) in the country is concerned. This is because many TB patients from these communities- remain under reached by the Revised National Tuberculosis Control Programme (RNTCP) of India. This marginalized community usually seeks early-stage healthcare from “friendly neighbourhood” non-formal health providers (NFHPs). Because, RNTCP has limited capacity to involve the NFHPs, an implementation research project was conceived, whereby an external partner would engage with the NFHPs to enable them to identify early TB symptomatics from this key sub-population who would be then tested using Xpert MTB/RIF technology. Diagnosed TB cases among them would be referred promptly to RNTCP for treatment. This paper aimed to describe the project and its impact.

**Methods:**

Adopting a quasi-experimental before-after design, four RNTCP units from two major urban-industrial areas of Odisha were selected for intervention, which spanned five quarters and covered 151,400 people, of which 30% were slum-dwelling migrants. Two similar units comprised the control population. The hypothesis was, reaching the under reached in the intervention area through NFHPs would increase TB notification from these traditionally under-notifying units.

RNTCP notification data during intervention was compared with pre-intervention era, adjusted for contemporaneous changes in control population

**Results:**

The project detected 488 Xpert^+^ TB cases, of whom 466 were administered RNTCP treatment. This translated into notification of additional 198 new bacteriologically positive cases to RNTCP, a 30% notification surge, after adjustment for 2% decline in control. This meant an average quarterly increase in notification of 41.20(20.08, 62.31; *p*<0.001) cases. The increase was immediate, evident from the rise in level in the time series analysis by 50.42(10.28, 90.55; *p* = 0.02) cases.

**Conclusion:**

Engagement with NFHPs contributed to an increase in TB notification to RNTCP from key under reached, slum-dwelling migrant populations.

## Introduction

Tuberculosis (TB) is considered a major public health problem in India, with almost 3 million people developing this infectious disease every year in the country, and as many as half a million Indians succumbing to it annually[1]. Despite country-wide implementation for more than a decade of the Revised National Tuberculosis Control Programme (RNTCP), which is based on the internationally acclaimed and recommended DOTS strategy, India is yet to reduce its TB burden substantially, let alone eliminate this dreadful scourge[1,2]. The basic tenets of RNTCP include diagnosing majority of the TB cases early in the course of their diseases and treating them appropriately and completely. This is to ensure that majority of the affected individuals suffer for a relatively shorter period and they do not continue to spread the infection to other uninfected individuals in the community. This has the potential to decelerate the infection-disease cycle, and subsequently reduce the TB burden in the society. For this, RNTCP primarily depends on voluntary presentation of the TB patients at the local, designated health facilities, which are overwhelmingly housed within the public health system of the country and very few within the private clinics.[3] Patients who initially seek care from private healthcare providers or health workers are also sometimes referred to the RNTCP designated facilities.

Individuals, who present for care at RNTCP facilities are then diagnosed by conventional sputum microscopy and also chest x-rays and other tests, if necessary, which are provided free of cost to the care-seekers. One of the major barriers to the decline of TB burden in India is some sections of its population remain largely “under reached”, at least in the initial stages of their diseases, to the programme[4,5]. In TB policy parlance, these groups are referred to as “key populations”—those with limited access to quality TB services, though the services are offered free by the national TB programmes[6] [7].

Migrant labourers constitute such an under reached key population in India[7,8]. These are the people, who mostly migrate from villages to the burgeoning Indian urban centres and its industrial patches. They mostly find employment in the low-skill, low-wage jobs in the informal sector, which has a sizeable presence in the Indian towns and cities. These marginalized people end up living in the overcrowded slums, the likes of which are rapidly proliferating in the urban and industrial landscape of modern India[9].

When individuals from this vulnerable group seek healthcare, they often find it difficult to navigate the channels of the public health system of their adopted places. This is mainly due to their unfamiliarity with the city health system. Moreover, the unsuitable location and timing of the public health facilities in these urban areas often impose substantial access and opportunity costs in the form of transportation costs and wage losses on these predominantly impoverished masses[10].

The high-end formal private healthcare system, which often has a large footprint in the Indian cities, is mostly out of reach of these poor people, because of their prohibitively steep user fees. Hence, the sick members of these poor, migrant communities often gravitate to the “friendly neighbourhood” non-formal health providers (NFHPs)[11], the likes of which include small medicine-shop owners, chemists and pharmacists of drug stores, practitioners of alternative systems of medicine, unqualified practitioners of allopathic medicine and members of various community-based health organizations. The NFHPs mainly prescribe medicines, mostly allopathic, for symptomatic relief of the care-seekers presenting to them. These NFHPs not only operate within or close to these slums, but also provide services at friendly hours and frequently offer flexi payment options[12] to suit the capacity of their poor clients. But, the lack of formal medical training of the NFHPs often lead to inappropriate management of grave medical conditions of their clients, thereby compounding their sufferings[13].

Naturally, those who develop TB in these communities—TB being considerably more common in urban India[1]- also often present initially to their neighbourhood NFHPS, frequently with symptoms of prolonged cough. These patients seek symptomatic relief or suggestions, early in the course of their diseases, when their TB symptoms are yet to become too severe to raise alarm in both the sufferers as well as their untrained providers. The NFHPS mostly provide inappropriate management to these care-seeking early TB symptomatics, which may include injudicious prescription of antibiotics also, as they are hardly aware of the diagnostic and treatment protocols of RNTCP [14]. This not only prolongs the sufferings of their TB patients by delaying their actual diagnosis and treatment initiation, as evidence shows[11], but also enables protracted transmission of infection in the community. This fuels the TB epidemic in these overcrowded environments[8]. However, some of these poor patients somehow ultimately manage to find their way to the RNTCP system[15], after much suffering, when their symptoms become too severe to ignore.

Unfortunately, the TB programmes worldwide [16], including RNTCP, hardly ever engaged with the NFHPS. The public-private-partnership initiatives of RNTCP were also mostly directed to the involvement of formally-trained medical practitioners, albeit many poor patients present to these NFHPs as their first “port-of-call”[17]. This insufficient engagement of RNTCP with this sizeable, yet important, non-formal healthcare sector has further been compounding the access issues of this key population, leading to many TB patients in these communities remaining under reached[16]. All these factors perhaps underpin the low notification of diagnosed TB cases from many Indian cities, notwithstanding a higher burden of TB in urban India, because many of these cities have substantially large under reached populations.

Therefore, a project was conceived whereby a local public health academic institute would facilitate establishment of linkage between the slum-dwelling migrant communities and RNTCP. The project planned to engage with the NFHPs on behalf of RNTCP, with an overarching aim to identify the TB patients emerging from these under reached communities through these providers and lead them to the standardized RNTCP regimen, as early in the course of their diseases as possible. The objective of our manuscript is to describe the project and assess its impact, using primarily the evaluation framework of the project and routine programme data, so that its successful components can be identified for future scale-up.

## Methods

### Populations and study design

Two intervention sites: Bhubaneswar city and Jajpur town in Odisha state were selected because these two areas are among those with the highest concentration of slum-dwelling migrant labourer population in the state—the primary target of the project. Bhubaneswar is the rapidly-growing capital city of this eastern Indian state. The industrial corridor of Jajpur district in Odisha harbours many large to small scale ferro alloy factories. These two intervention sites are similar in nature in terms of migrant labourers residing in slums that dots these two areas extensively as well as the care-seeking practices of these communities. The total population of the intervention area hereinafter is called the Evaluation Population (EP)

The control area that did not receive any intervention from the project but was only used for comparison, consisted of Cuttack city and its outskirts. Cuttack, a city located 25 kilometres away from Bhubaneswar, shares similar characteristics with the intervention area. It being the commercial hub of the state, also traditionally attracts many migrant labourers to the numerous commercial and industrial establishments situated in and around the city, as well as the informal service sector allied to them. Therefore, many slums emerge and continue to proliferate also in Cuttack and its outskirts, where these migrant communities reside. The total population of the control area is hereinafter referred to as the Control Population (CP). Historically, both the intervention and the control areas experienced low TB case notification to RNTCP—notification failing to attain 50% of the estimated annual incident cases, let alone the prescribed 90% needed to effect a significant decline of TB[18].

The demographic and other relevant characteristics of the intervention and control areas are described in Table 1.

**Table 1:** The characteristics of intervention and control areas.

	Intervention area		Control area
	Bhubaneswar site	Jajpur site	Cuttack city and outskirts
Number of Basic Management Units (BMUs) providing TB services	2	2	2
Number of designated microscopy centres providing TB diagnostic services	4	4	6
Number of peripheral health institutions or treatment centres providing RNTCP treatment services	6	6	8
Total population (2014)*	850,000	664,000	1,411,000
Proportion of total population who are mainly migrant labourers (dwelling in slums in these areas)^$^	Approximately 30%	Approximately 30%	Approximately 35%
Average baseline new smear positive case notification rate per 100,000 population per year (1st quarter 2011 to 3rd quarter 2014)^¶^	37.4	41.2	19.7

* The total population of the intervention area is known as the evaluation population—EP (1,514,000) and that of the control area is known as the control population—CP (1.411,000)

^$^ This was the target population of the project in the intervention area

^¶^ The estimated new smear positive case incidence is 85 per 100,000 population per year. So the baseline annualized new smear positive case detection rate (case notification/estimated incidence) was <50% in all the areas

We used a quasi-experimental before-after design with a control group to analyse the results of intervention, which is frequently employed to evaluate such population-level initiatives or events[19][20].

### Intervention

In addition to the routine passive case detection services provided by RNTCP the project engaged in the intervention area with almost all the Non-formal Health Providers (NFHP) who were embedded within and were providing services to the target population. The project was administered by Asian Institute of Public Health, a public health academic institute based in Bhubaneswar, Odisha. The project enlisted (n = 539), enrolled (n = 447) and then engaged with the NFHPs (n = 431) from the target population, in September 2014. The project staff, experienced in working with community-based organizations and providers, used persuasion and one-to-one personal engagement with the NFHPs to motivate them to participate in the project. The NFHPS were trained by the project staff to identify the presumptive TB cases (also known as TB symptomatics) from their clientele using presence of cough for two weeks or more as the primary screening criterion as suggested in the Technical Guidelines for RNTCP. The NFHPs were also trained to collect quality sputum samples from these symptomatics, promptly, either at their clinics or at the patients’ homes. The NFHPs were also provided supportive supervision by the project staff, at regular intervals, either through personal visits or telephonic contacts, to monitor the quality of the operations and also to sustain their morale. No NFHP dropped-out from the project.

The field network of the project transported the samples collected from the NFHP-identified suspects to two project laboratories, one established at each intervention site. Both laboratories were equipped with Xpert MTB/RIF technology, a cartridge based nucleic acid amplification (CBNAAT) test with >90% sensitivity and specificity[21,22]. This technology simultaneously detects *Mycobacterium tuberculosis* and resistance to the most important first-line anti-TB drug—Rifampicin (RIF), from just one sample of sputum, in less than 2 hours. This is in stark contrast to the routine sputum smear microscopy—the primary diagnostic test used by RNTCP, which requires TB symptomatics to make at least two visits, on two different days, to the designated RNTCP facilities for diagnosis and which is only 50% sensitive[23,24].

One Xpert MTB/RIF laboratory was set up at the largest government hospital of Bhubaneswar city and the other one was hosted by a health dispensary, operated by the steel industry for the local community in the industrial area of Jajpur district.

**Fig 1:**
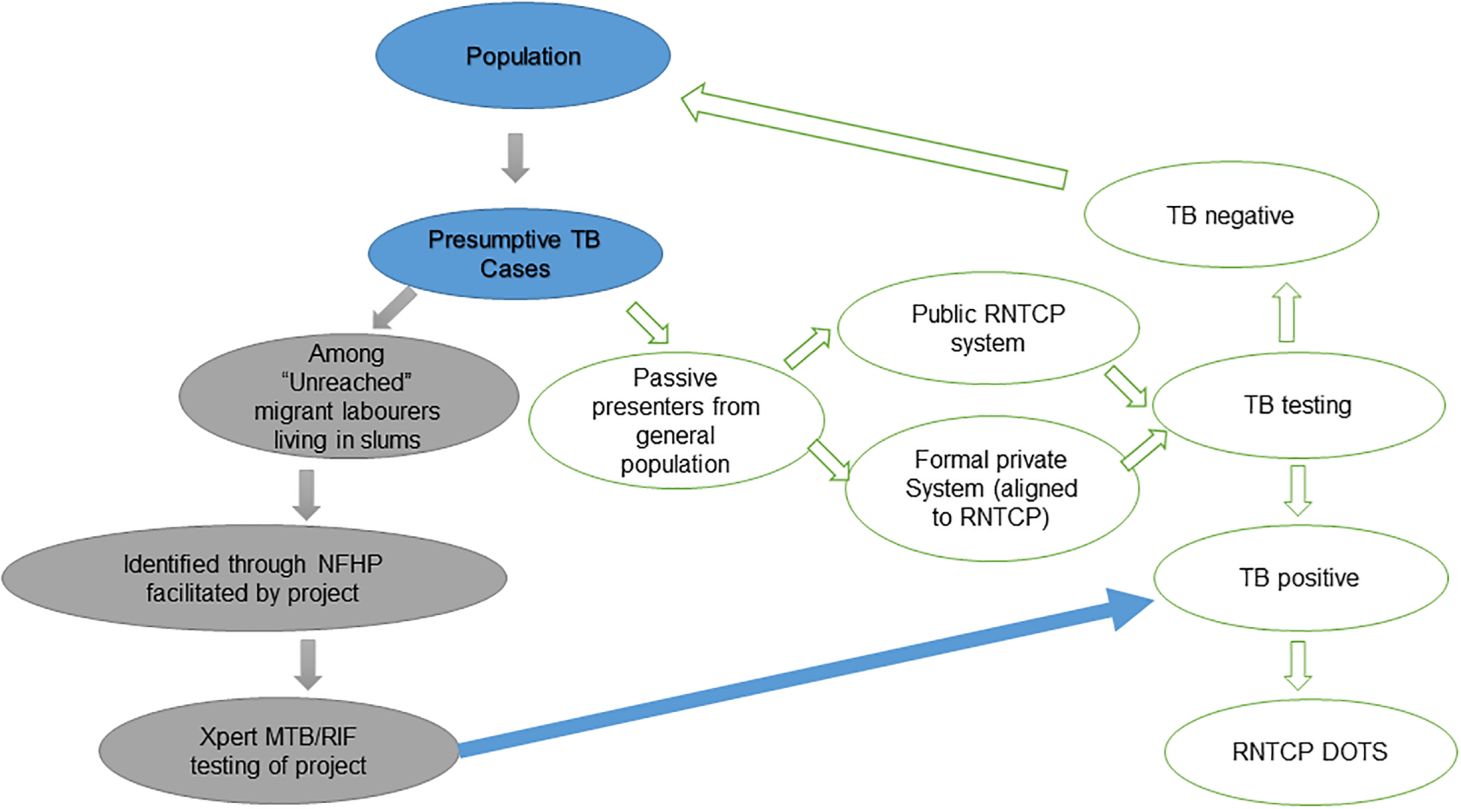
Schematic presentation of flow of target patients through the project and RNTCP system.

Those symptomatics, screened by the NFHPs, who turned out to be positive to Xpert test (Xpert^+^), were then supported by the project staff to promptly register for standard anti-TB treatment from the nearest RNTCP health facility. The information of their diagnosis was also conveyed to those NFHPs who initially had identified them. The project staff also facilitated retrieval of the few diagnosed patients who failed or refused to attend the RNTCP clinics for treatment initiation. Fig 1 depicts the flow of the patients through the project and RNTCP.

These Xpert^+^ TB patients, identified by the project, were notified to the local Basic Management Units (BMUs) of RNTCP (Table 2). These cases were then included in the quarterly RNTCP reports of BMU and therefore in the district and state quarterly RNTCP notification reports. The project was implemented for five quarters, from 4^th^ quarter 2014 to 4^th^ quarter 2015.

**Table 2:** The yield of the project.

Indicators	Numbers
Number of patients checked/enquired of TB symptoms by the NFHPs in the evaluation population	253679
Number (%) of TB symptomatics identified	3780 (1.49%)
Number (%) of TB symptomatics examined for TB using Xpert RIF/MTB	2800 (74%)
Number (%) of TB symptomatics examined for TB confirmed as Xpert^+^	488 (17.4%)
Number (%) of Xpert^+^ (RIF Sensitive) patients started on RNTCP treatment	466 (95.5%)

The control population did not receive any intervention from the project and continued to receive only routine RNTCP services, which meant the NFHPs of that area were uninvolved in RNTCP.

The testable hypothesis of this project was that if this initiative through NFHP involvement succeeded to bring these under reached TB patients within the fold of the programme, then RNTCP case notification rate would increase significantly during the intervention period in the evaluation population, as compared to that in the control population.

### Data

Number of individuals screened by the NFHPs, number of symptomatics identified among them, number of symptomatics undergoing Xpert MTB/RIF examination and numbers of Xpert^+^among those examined were collected from the laboratory records of the project. The monthly aggregates of these results were also sent to the RNTCP management information system, every month, for integration with the routine RNTCP microscopy services data.

The data of the Xpert^+^ patients, those who registered for RNTCP treatment, were also integrated with the routine RNTCP case notification data at the BMU level as mentioned above. The number of cases notified by the project marked its yield. However, the impact of the project was assessed by the additional cases notified by RNTCP during the intervention period as compared to the notification data of previous year(s). The new bacteriologically positive (bac^+^) cases, which was primarily used to assess the impact, included newly diagnosed Xpert ^+^ cases from the project plus the routine sputum smear positive cases of RNTCP. Total cases included bacteriologically positive, bacteriologically negative but x-ray positive and extra-pulmonary cases of both new and retreatment types. Quarterly case notification data for both bac^+^ and total cases were collected from the routine RNTCP reports of the BMUs—both from the evaluation and control populations. Few bac^+^ cases detected by the project were not new but of retreatment category. They were not included in the analysis of impact as per the project monitoring and evaluation guidelines.

Population data of the BMUs were collected from the RNTCP reports that used census figures.

The data used for the`analyses were BMU aggregates that were extracted in a completely de-identified format from the routine quarterly RNTCP reporting system. No individual-level data was collected from the patients or used in the analyses. Hence, there was no necessity to acquire informed consents or anonymize the data further.

### Statistical analysis

We first used descriptive statistics to report the yield of the project.

We then conducted the impact analysis of the project based on additional new bac^+^ cases notified by the project, using various methods.

**Fig 2:**
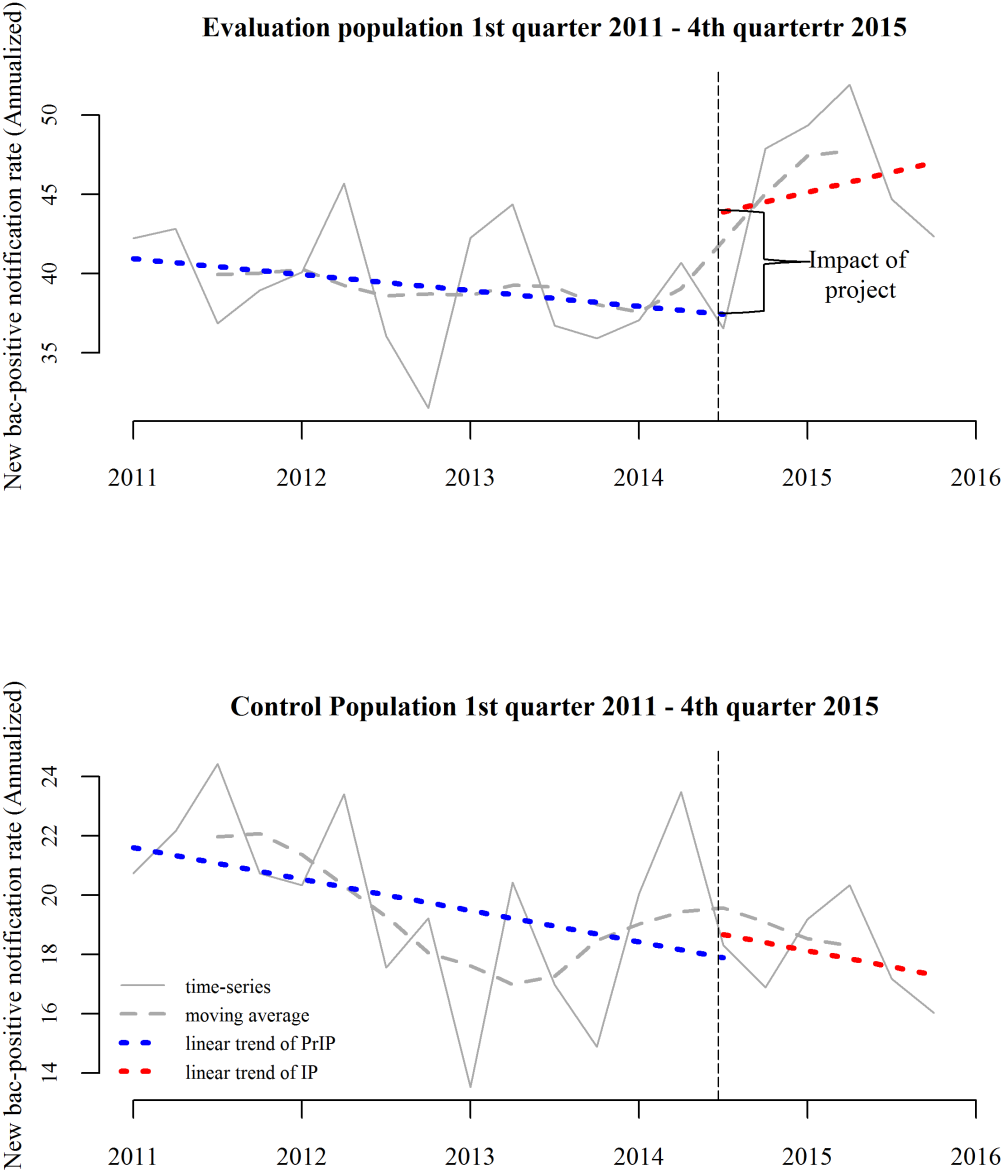
Interrupted time series analysis of new bac+ cases.

1. New bac+ cases notified during the five quarters of project implementation period, hereinafter referred to as the “intervention period” (IP), was compared with the cases notified in the five quarters of “pre-intervention period” (PrIP). The five quarters of IP spanned from 4^th^ quarter 2014 (starting from 1^st^ October 2014) to 4^th^ quarter 2015 (ending at 31^st^ December 2015).
  The notification data of 4^th^ quarter 2013 to 3^rd^ quarter 2014 plus the notification data of 4^th^ quarter 2013 added again made up the PrIP notification figures. The data of 4^th^ quarter 2013 was added twice to make up the five quarters of PrIP. This was done to account for seasonality, so that the five quarters of PrIP closely matched those of IP.
  The sum of new bac+ cases notified during the five quarters of IP minus the sum of new bac+ cases notified during the five quarters of PrIP gave us the unadjusted increase in case notification in the evaluation population. We estimated the change in notification in the control population during the same period. The change in notification in the evaluation population minus the change in notification in the control population gave us the control-adjusted increase in case notification in the evaluation population. This control-adjusted increase is referred to as the additional cases—the primary metric used to study the impact of intervention. The same approach was also adopted for total cases notified (see above for definition of total case).
2. Next, we used an Ordinary Least Square (OLS) regression framework to estimate the changes in the quarterly average notification of new bac+ cases between the IP and PrIP, both in the evaluation as well as control population. This was done only for new bac+ cases as these were the primary focus of the project. Then we estimated by how much the average changes varied between evaluation and control population: the difference-in-difference (DD) estimator (Eq 1). The OLS framework allowed us to test statistical significance of the DD estimates conveniently.
  $$Y=\beta_{0}+\beta_{1}\left(pop\right)+\beta_{2}\left(time\right)+\beta_{3}\left(pop*time\right)+\varepsilon$$ (1)
  Where *Y*: Average new bac+ cases notified/notification rates; pop: dummy variable representing evaluation population or control population; *time*: dummy variable representing IP or PrIP; β0: intercept; β1: mean difference in notified new bac+ cases/rates between evaluation population and control population; β2: mean difference in notified new bac+ cases between IP and PrIP; β3: DD estimate—the parameter of interest; ϵ: error
  The DD estimation used five quarters of IP data and five quarters of PrIP data. Additionally, five quarters of IP data and fifteen quarters of PrIP (1^st^ quarter 2011 to 3^rd^ quarter 2014) data were also considered in a separate estimation model. The models were executed with absolute numbers of cases notified as well as annualized case notification rates/100,000 population
3. DD estimate is based on a “parallel trend” or “common trend” assumption, which assumes that in the absence of any intervention the new bac+ case notification trends in the evaluation population would have followed that of the control population. This assumption could not be tested using the methodology described in (1), where we used the averages of notification of five quarters each, from both IP and PrIP, not accounting for the prior trends. Since the violation of this assumption can substantially bias the estimates[20], we examined prior trends for previous fifteen quarters (as mentioned above). The visual examination showed very little difference between the evaluation and control population in terms of prior notification trends (Fig 2). Additionally, we also conducted interrupted time-series analysis with a comparison group, using segmented linear regression[25] (Eq 2), whereby the differences between the evaluation and control population were estimated in terms of their pre-post differences in slopes (signifying long-term effect of intervention) and intercepts (signifying immediate effect of intervention) (Eq 2).
  $$Y_{t}=\beta_{0}+\beta_{1}T_{t}+\beta_{2}X_{t}+\beta_{3}X_{t}T_{t}+\beta_{4}Z+\beta_{5}ZT_{t}+\beta_{6}ZX_{t}+\beta_{7}ZX_{t}T_{t}+\varepsilon_{t}$$ (2)
  Y_t_: new bac+ cases notified/notification rates at equally-spaced time-point_t_ i.e. quarters; T_t_: identifiers of quarters; X_t_: dummy variable representing IP or PrIP; Z: dummy variable representing evaluation or control population. Parameters of interest included β_6_ that represented the estimator of difference in level changes, marking the “immediate effect” and β_7_ that represented the estimator of difference in slope changes.

95% confidence intervals (CI) of all the parameter estimates were constructed and their statistical significances tested at 5%. The analysis was conducted using R statistical software[26] (version 3.3.0)

### Sensitivity analysis

We repeated the analysis (2) with eleven PrIP quarters instead of fifteen PrIP quarters, as it may be argued that our choice of fifteen quarters was somewhat arbitrary.

### Ethical approval

Clearance for implementation of the project was received from State TB Cell, Department of Health and Family Welfare, Government of Odisha, dated 28th January 2014. Letter No. 136/TB cell

## Results

### Yield

The NFHPs checked 256,379 clients for TB symptoms, in the evaluation population, during the five quarters of intervention, of which approximately 1.5% were TB symptomatics cases (n = 3780). Of all the symptomatics, 74% could be subjected to Xpert testing under the project, out of whom 488 (17.4%) were diagnosed as Xpert^+^ cases. Ninety five percent of these Xpert^+^ cases (n = 466) could be registered for RNTCP treatment in the local BMUs covering the evaluation population (Table 2). Only three of these 488 bac^+^ patients tested positive for rifampicin resistance and they were referred to the designated drug-resistant TB treatment centre of RNTCP for further management.

### Additional cases notified

During the five quarters of intervention period (IP) there was an increase in notification of 198 new bac+ cases as compared to five quarters of pre-intervention period (PrIP). A 28% unadjusted increase in new bac+ case notification, and after accounting for 2% decline in notification in the control population during the same period, a control-adjusted 30% increase in notification was registered by the intervention area. For total cases that include all types of cases the corresponding increase was 192 against the backdrop of an increase of 198 new bac^+^ cases. This signifies that except new bac^+^ cases, which registered an increase in notification, the other types of TB cases, for instance smear negative, extra-pulmonary and retreatment cases, collectively registered a marginal decline of 6 cases during the intervention period.

The control-adjusted DD estimate for the same period was 41.20 (20.68, 62.31), which signifies the average quarterly increase in notified cases in the evaluation population after adjusting for changes in control population (Table 3).

**Table 3:** Differences in additional new bac+ cases and total cases notified (five quarters of PrIP vs five quarter of IP) by the evaluation population (evaluation population) and control population (control population) and the difference-in-difference estimates.

	Change in notified cases in the Evaluation Population n (%)	Changes in notified cases in the Control Population n (%)	DD estimate (95% CI, p value)
New bac+ cases	199 (28%)	-7 (-2%)	41.20 (20.08, 62.31; *p*<0.001)
Total cases	192 (10%)	89 (8%)	20.60 (-12.86, 54.06; *p* = 0.21)

When compared with 15 quarters of PrIP, the control-adjusted DD estimates for quarterly average change of absolute notification and notification rates were 41.26 (18.83, 63.68, p<0.001) and 9.86 (4.05, 13.30, p<0.001) respectively.

The results of segmented regression of the interrupted time-series of notification rates are illustrated in Fig 2. Almost parallel prior trends in PrIP, though with different levels, could be observed. In the evaluation population, a substantial increase in the level was observed immediately after the intervention was introduced—the so called “immediate effect”. There was no such change in the control population. The estimate of the immediate effect in terms of absolute notification was 50.42 (10.28, 90.55) and the notification rate was 13.36/100,000 population (2.78, 23.93) (Table 4). However, the immediate change was not accompanied by any significant longer term changes in trends. (Table 4)

**Table 4:** Results of interrupted time series analysis of new bac+ cases notified and notification rates/100,000 population.

Indicators	Estimate (numbers) (95% CI; *p* value)	Estimate (rates) (95% CI; *p* value)
Change in level ("immediate effect") in the Evaluation Population	52.72 (18.85, 86.58); *p* = 0.007*	14.51 (6.07, 22.94); *p* = 0.003*
Change in slope in the Evaluation Population	-6.11 (-16.29, 2.53); *p* = 0.17	-1.32 (-3.66, 1.02); *p* = 0.28
Change in level ("immediate effect") in the Control Population	2.30 (-19.23, 23.83); *p* = 0.83	1.14 (-5.23, 7.52); *p* = 0.72
Change in slope in the Control Population	-0.38 (-6.36, 5.59); *p* = 0.91	-0.10 (-1.87, 1.66); *p* = 0.91
Estimate of difference in level changes ("Immediate effect")	50.42 (10.28, 90.55); *p* = 0.02*	13.36 (2.78, 23.93); *p* = 0.02*
Estimate of difference in slope changes	-6.49 (-17.64, 4.65); *p* = 0.26	-1.21 (-4.14, 1.72); *p* = 0.42

*statistically significant

### Sensitivity analysis

The parameter estimates of interest hardly changed when the PrIP period was shortened to eleven quarters from fifteen. The control-adjusted DD estimates for quarterly average change of absolute notification and notification rates were 41.13 (18.14, 64.37, p<0.001) and 9.42 (3.93, 14.70, p = 0.0015) respectively in the sensitivity analysis.

## Discussion

There was an increase in new bacteriologically positive case notification from the evaluation population by 198 cases during the five quarters of intervention—a 30% increase from the corresponding five quarters prior to intervention. The difference-in-difference estimates showed an increase of approximately 41 cases, on an average, for each quarter of intervention, in the evaluation population. This estimate remained consistent when five quarters of intervention was compared either with five quarters or fifteen quarters of pre-intervention era. Although, the control population was similar to the evaluation population, during this period it showed a marginal decline (2%) in new bac+ case notification. These two trends imply that the surge in new bac+ case notification in the evaluation population during the intervention period was largely attributable to the intervention. This increase of notification in the evaluation population was achieved against the backdrop of slow decline of notification in the pre-intervention period, both in the evaluation and control population. This indicates that the notification trend in the evaluation population was significantly bucked, that too almost “immediately”, as soon as the project was implemented, confirmed by the significant changes in the level in the interrupted time-series analysis (Fig 2). It cannot be entirely ruled out that there might have been contributions to this surge from other intensified case-finding initiatives in the evaluation population being implemented during that period, albeit anecdotally there is no such evidence of that. Therefore, the broad conclusion that the surge in the case notification in the evaluation population was mainly due to the intervention project, will not be inaccurate.

This increase in case notification was achieved by the project through inclusion and involvement of the non-formal health providers (NFHP) within the operational framework of RNTCP. This is because the target population of the project that is the slum-dwelling migrant labourer population living in Bhubaneswar and Jajpur industrial areas of Odisha, presented to these providers in large numbers and not to the public health system. Therefore, as the project implementers functioned as a go-between the RNTCP and the NFHP network, it facilitated early and convenient access of the TB patients from the target population to standardized TB care of RNTCP, through the NFHPs, resulting in the observed increase in case notification

The project is likely to have benefitted considerably from the use of Xpert MTB/RIF technology as the first line of test in its diagnostic algorithm—a divergence from the currently existing RNTCP policy[3]. Consequently, the bacteriologically positive case pick-up rate from the TB symptomatics examined by the project was 17%, as often found in such projects employing Xpert MTB/RIF as the first-line of diagnostic test [27–29]. This was considerably higher than that achieved by the routine programme tools i.e. smear microscopy, the positivity rate of which is roughly 10% in Odisha. Moreover, the short turnaround time taken to diagnose cases through this technology also helped to avoid significant loss of diagnosed TB cases from the treatment system. However, similar projects using Xpert MTB/RIF as a primary diagnostic tool, instead of sputum microscopy, have been found to increase the notification of more bacteriologically confirmed TB cases, but without enhancing the notification of total TB cases[29] [27,28,30]. The reason is many such projects fail to tap into the pool of under reached populations; but just tend to bacteriologically confirm through the use of Xpert MTB/RIF technology, the sputum negative TB cases—those who anyway would have been diagnosed by X-ray or empirically[31] by the programme. Therefore they just tend to shift some bacteriologically-unconfirmed cases to the bacteriologically-confirmed category and therefore only increase notification of positive cases but fail to impact total notification. However, additional 198 bacteriologically-confirmed cases notified by this project led to an increase in notification of total patients by 192. This indicates that the additional new bac+ case notification, attributed to the project intervention, were actually from the under reached population, who otherwise would not have been diagnosed, at least during that time period, if the project was not implemented.

National TB programmes, including RNTCP, will eventually strengthen their country-wide diagnostic network with Xpert MTB/RIF technology. However, just replacing routine smear microscopy by more sensitive molecular technology may not help the programme to diagnose majority of the TB cases from the key under reached populations, as barriers to access will continue to plague routine, passive case finding strategies. To reach out to these key populations, which is central to TB elimination strategy of India, external community-based programme partners may be needed to establish the all-important connections between RNTCP and the vulnerable communities and their community-based healthcare providers[32], as this project has demonstrated. Recent mathematical models have also shown that involvement of private healthcare sector, both formal and informal, among all other intervention strategies, will have maximum impact on TB control especially if complemented by large scale roll-out of Xpert MTB/RIF across the country[32][33].

The analyses also illustrates some weaknesses in the project initiative and also perhaps in the impact-evaluation framework[34]. We observe that 466 Xpert^+^ positive cases were diagnosed by the project and facilitated by it to register for RNTCP treatment; but only 198 (43%) cases were actually additional new bac+ cases among them. It illustrates that perhaps more than half of the Xpert^+^ cases detected by the project would have anyway accessed RNTCP and would have been detected by routine smear microscopy or chest x-ray. This was further evident from the post-intervention trends, which experienced a sharp immediate rise in case notification, but then the slope flattened, which means further increase after the initial surge could not be sustained. Moreover, the case detection could only be increased to 55%, from the baseline 42%, of the estimated incident cases—indicating perhaps an unfulfilled potential of the project. This was most likely due to inability of the project to quickly move on to new under reached populations, living in untapped slums. If that could have been done, a greater number of under reached population could have been reached and more additional TB cases could have been detected with the same project yield, over the same period. Some of these weakness may be ascribed to lack of time, as the project was implemented for five quarters only. Therefore, the project failed to quickly manoeuvre from one slum to another.

Secondly, some of the cases detected by the project were of retreatment category, but since the entire impact evaluation framework was focused on the additional notification of new cases only, additional notification in the retreatment category was not considered—perhaps a missed opportunity to apportion some of this credit to the project.

Another gap in the project was 26% of the TB symptomatics identified by the NFHPs could not be subjected to sputum examination and 5% of the TB cases diagnosed by the project could not be initiated on RNTCP treatment. Dropping out of the diagnosis and treatment system of TB programmes by a proportion of symptomatics and patients is not uncommon. The target population of the project being migrant in nature, few of them failed to comply with its diagnosis and treatment protocols despite the persuasion by the providers and the project staff. Otherwise, the impact of the project could have been more with the same effort. Some of the “initial defaulters”–those who failed to initiate treatment after diagnosis—may have ended up receiving RNTCP treatment from their original places of residence, but the project had no means to reconcile that information. However, the proportion of initial defaulters encountered in the project was less than what has been the overall experience of RNTCP in these areas.

The analysis has the usual limitations as well the strengths of implementation research using quasi-experimental design. The control population was perhaps not an exact match of the evaluation population, though the similarity was remarkable as described above. However, the case notification from the control area had historically been even lower than that of the evaluation population. Therefore, had there been any state-wide initiative from the routine RNTCP system to enhance case notifications, it would have had greater impact on the control population due to its lower base. But, in reality the control area showed a slight decline in notification during the intervention period, in stark contrast to the 28% increase in notification in the intervention area. Additionally, the before-after design and the difference-in-difference analysis framework also addressed some of these unmeasured differences, if any, robustly enough[25]. The interrupted time series analysis with a comparison group also validated the parallel trend assumption of this design and also helped to identify the timing of the impact of the project. The minimum change in the sensitivity analysis further strengthened our analytical approaches. Finally, we did not conduct any economic evaluation of the project as well as assessment of delay in diagnosis. That would have thrown light into the cost and burden-reduction implications of the initiative, which could have strengthened its future scale-up potentials.

In spite of all the weaknesses, results from our project provide robust evidence that case detection in key slum-dwelling, migrant labourer populations can be increased through involvement of non-formal providers and that too within a short period. Such efforts of involving NFHPs exclusively, of which there are few other evidences from India and similar settings [35][33], is very much aligned to the Global Plan to End TB of the Stop TB Partnership. It is likely that, if it is scaled-up, it may help RNTCP to reach a large section of the under reached populations in the urban slums, and bend the TB epidemic curve in the country by helping to severe the infection-disease cycle in such overcrowded environments. This may enable the country to achieve the key objectives of WHO’s End TB Strategy of 2015[36]. The results of the project were obtained in routine field conditions, hence they may be replicable, at least in similar urban and industrial-area settings. The simple design of this intervention also underscores its wide scalability across different geographical regions. Studies of “patent medicine vendors” of Nigeria[37], involvement of rural informal doctors in Bangladesh[38] and models of informal provider involvement in West Bengal demonstrate advantages of mainstreaming the NFHPs with defined roles[13], but little evidence exists from their exclusive involvement around urban slums and in TB control, albeit some evidence from other disease-control domains exist[39]. This signifies the importance of our work as a template for such similar, future anti-TB initiatives.

To conclude, this work presents a proof of concept that by involving the community-embedded non-formal health providers, through external partners, under reached cases from the key migrant labourer populations, living in the slums, can be diagnosed quickly in numbers. Scaling-up of such models in areas with such large key vulnerable populations may be a way forward for the programme to diagnose majority of the cases from these underserved communities. This will not only reverse the TB epidemic in those sub-populations and hence the entire country[33], but also protect this marginalized community from far-reaching, unfavourable economic consequences of TB[40].
